# Supplementary material for: It’s all in the music: A systematic review on the effects of musical characteristics on participants’ experience and behavior during leisure activities
Source: PLoS One. 2025 Jul 22;20(7):e0315986. doi: 10.1371/journal.pone.0315986 (PMC12282921; doi:10.1371/journal.pone.0315986)
Supplement: S1 File — Includes search strings for the following databases: PubMed, Cochrane, Embase Elsevier, and PsychInfo. (DOCX) [file pone.0315986.s001.docx]

## **Search string PubMed**

((“music venue*”[Title/Abstract]) OR (nightclub*[Title/Abstract]) OR (disco*[Title/Abstract]) OR (concert*[Title/Abstract]) OR (pub[Title/Abstract]) OR (bar[Title/Abstract]) OR (festival[MeSH Terms]) OR (festivals[MeSH Terms]) OR (activities, leisure[MeSH Terms]) OR (activity, leisure[MeSH Terms]) OR (“leisure activit*”[Title/Abstract]))

AND

((music[MeSH Terms]) OR (music* [Title/Abstract]) OR (“noise induced”[Title/Abstract]) OR (hearing loss, noise induced[MeSH Terms]) OR (noise induced tinnitus[MeSH Terms]))

AND

((boredom[MeSH Terms]) OR (frustration[MeSH Terms]) OR (irritable mood[MeSH Terms]) OR (behavior, drug seeking[MeSH Terms]) OR (anger[MeSH Terms]) OR (alcohol drinking[MeSH Terms]) OR (personal satisfaction[MeSH Terms]) OR (pleasure[MeSH Terms]) OR (happiness[MeSH Terms]) OR (loneliness[MeSH Terms]) OR (sexual behavior[MeSH Terms]) OR (euphoria[MeSH Terms]) OR (behavior[MeSH Terms]) OR (behavior control[MeSH Terms]) OR (verbal behavior[MeSH Terms]) OR (social behavior[MeSH Terms]) OR (social behaviors[MeSH Terms] ) OR (behavior, obsessive[MeSH Terms]) OR (mass behavior[MeSH Terms]) OR (dangerous behavior[MeSH Terms]) OR (adolescent behavior[MeSH Terms]) OR (violence[MeSH Terms]) OR (smoking[MeSH Terms]) OR (behavior*[Title/Abstract]) OR (behaviour*[Title/Abstract]) OR (“alcohol drinking”[Title/Abstract]) OR (smoking[Title/Abstract]) OR (experience[Title/Abstract]))

**Search string Cochrane**

#1 MeSH descriptor: [Boredom] explode all trees

#2 MeSH descriptor: [Frustration] explode all trees

#3 MeSH descriptor: [Irritable Mood] explode all trees

#4 MeSH descriptor: [Illicit Drugs] explode all trees

#5 MeSH descriptor: [Alcohol Drinking] explode all trees

#6 MeSH descriptor: [Alcohol Abstinence] explode all trees

#7 MeSH descriptor: [Anger] explode all trees

#8 MeSH descriptor: [Personal Satisfaction] explode all trees

#9 MeSH descriptor: [Pleasure] explode all trees

#10 MeSH descriptor: [Happiness] explode all trees

#11 MeSH descriptor: [Euphoria] explode all trees

#12 MeSH descriptor: [Loneliness] explode all trees

#13 MeSH descriptor: [Love] explode all trees

#14 MeSH descriptor: [Behavior] explode all trees

#15 (behavior*):ti,ab,kw OR (response):ti,ab,kw OR (experience):ti,ab,kw OR (behaviour*):ti,ab,kw OR (“alchohol drinking”):ti,ab,kw (Word variations have been searched)

#16 (smoking):ti,ab,kw

#17 #1 OR #2 OR #3 OR #4 OR #5 OR #6 OR #7 OR #8 OR #9 OR #10 OR #11 OR #12 OR #13 OR #14 OR #15 OR #16

#18 MeSH descriptor: [Leisure Activities] this term only

#19 (nightclub*):ti,ab,kw OR (festiv*):ti,ab,kw OR (bars):ti,ab,kw (Word variations have been searched)

#20 #18 OR #19

#21 MeSH descriptor: [Music] this term only

#22 MeSH descriptor: [Sound] this term only

#23 MeSH descriptor: [Noise] this term only

#24 MeSH descriptor: [Tinnitus] this term only

#25 MeSH descriptor: [Hearing Loss, Noise-Induced] this term only

#26 (music*):ti,ab,kw OR (sound):ti,ab,kw OR (noise*):ti,ab,kw (Word variations have been searched)

#27 #21 OR #22 OR #23 OR #24 OR #25 OR #26

#28 #17 AND #20 AND #27

## **Search string Embase Elsevier**

#1 ‘music’ /mj OR ‘sound’ /mj OR ‘noise’ /mj OR ‘noise injury’ /mj OR ‘music*’ :ti,ab OR ‘sound’ :ti,ab OR ‘noise*’ :ti,ab

#2 ‘leisure’ /mj OR ‘nightclub*’ :ti,ab OR ‘concert’ :ti,ab OR ‘bars’  OR ‘festival*’ :ti,ab OR ‘leisure activit*’ :ti,ab

#3 ‘anger’/de OR ‘boredom’/de OR ‘disgust’/de OR ‘euphoria’/de OR ‘frustration’/de OR ‘happiness’/de OR ‘mental irritation’/de OR ‘mood’/de OR ‘pleasure’/de OR ‘unhappiness’/de OR ‘irritability’/de OR ‘temperament’/exp/mj OR ‘illicit drug’/de OR ‘drinking behavior’/mj OR ‘sexual behavior’/mj OR ‘adolescent behavior’/de OR ‘aggression’/de OR ‘antisocial behavior’/de OR ‘help seeking behavior’/de OR ‘masochism’/de OR ‘mass behavior’/de OR ‘reward seeking behavior’/de OR ‘substance use’/exp OR ‘response’:ab,ti OR ‘experience’:ti,ab OR ‘behavior*’:ab,ti OR ‘behaviour*’:ab,ti OR ‘alcohol drinking’:ab,ti OR ‘smoking’:ab,ti

#1 AND #2 AND #3

## **Search string PsychInfo**

((**Index Terms**: ("music")) *OR* (**Index Terms**: ("Music Perception")) *OR* (**Index Terms**: ("Auditory Perception")) *OR* (**Index Terms**: ("Auditory Stimulation")) *OR* (**Index Terms**: ("Noise Effects")) *OR* (**title**: ("sound level")) *OR* (**title**: (music*)) *OR* (**abstract**: (music*)) *OR* (**title**: (sound)) *OR* (**abstract**: (sound)) *OR* (**title**: (noise)) *OR* (**abstract**: (noise)) *OR* (**title**: ("noise induced")) *OR* (**abstract**: ("noise induced")) *OR* (**abstract**: ("sound level"))) *AND* ((**abstract**: ("music venue")) *OR* (**title**: ("music venue")) *OR* (**title**: (nightclub*)) *OR* (**abstract**: (nightclub*)) *OR* (**title**: (disco*)) *OR* (**abstract**: (disco*)) *OR* (**title**: (concert*)) *OR* (**abstract**: (concert*)) *OR* (**title**: ("pub")) *OR* (**abstract**: ("pub")) *OR* (**title**: ("bar")) *OR* (**abstract**: ("bar")) *OR* (**title**: (festiv*)) *OR* (**abstract**: (festiv*)) *OR* (**title**: ("leisure activit*")) *OR* (**abstract**: ("leisure activit*"))) *AND* ((**title**: (behavior*)) *OR* (**Index Terms**: ("Boredom")) *OR* (**Index Terms**: ("agitation")) *OR* (**Index Terms**: ("anger")) *OR* (**Index Terms**: ("contentment")) *OR* (**Index Terms**: ("happiness")) *OR* (**Index Terms**: ("pleasure")) *OR* (**Index Terms**: ("satisfaction")) *OR* (**Index Terms**: ("Dissatisfaction")) *OR* (**Index Terms**: ("Disappointment")) *OR* (**Index Terms**: ("Distress")) *OR* (**Index Terms**: ("Euphoria")) *OR* (**Index Terms**: ("Irritability")) *OR* (**Index Terms**: ("Extraversion")) *OR* (**Index Terms**: ("Sexuality")) *OR* (**Index Terms**: ("Sociability")) *OR* (**Index Terms**: ("Alcohol Intoxication")) *OR* (**Index Terms**: ("Blood Alcohol Concentration")) *OR* (**Index Terms**: ("Binge Drinking")) *OR* (**Index Terms**: ("Drug Seeking")) *OR* (**Index Terms**: ("Drug Abuse")) *OR* (**Index Terms**: ("Drug Addiction")) *OR* (**Index Terms**: ("Drug Dependency")) *OR* (**Index Terms**: ("Aggressive Driving Behavior")) *OR* (**Index Terms**: ("Aggressive Behavior")) *OR* (**Index Terms**: ("Adolescent Behavior")) *OR* (**Index Terms**: ("Attack Behavior")) *OR* (**Index Terms**: (Drinking Behavior)) *OR* (**Index Terms**: ("Social Behavior")) *OR* (**Index Terms**: ("Tobacco Smoking")) *OR* (**abstract**: (behavior)) *OR* (**title**: (behavior)) *OR* (**abstract**: (behaviour*)) *OR* (**title**: (behaviour*)) *OR* (**title**: ("alcohol drinking")) *OR* (**abstract**: ("alcohol drinking")) *OR* (**title**: (smoking)) *OR* (**abstract**: (smoking)) *OR* (**title**: (experience)) *OR* (**abstract**: (experience)))
