# Supplementary material for: It’s all in the music: A systematic review on the effects of musical characteristics on participants’ experience and behavior during leisure activities
Source: PLoS One. 2025 Jul 22;20(7):e0315986. doi: 10.1371/journal.pone.0315986 (PMC12282921; doi:10.1371/journal.pone.0315986)
Supplement: S2 File — (DOCX) [file pone.0315986.s002.docx]

## **NOS for cohort studies**

Selection

- Representativeness of the exposed cohort
- Selection of the non-exposed cohort
- Ascertainment of exposure
- Demonstration that outcome of interest was not present at the start of the study

Comparability of cohorts on the basis of the design or analysis (worth 1 or 2 stars)

Outcome

- Assessment of outcome
- Was follow-up long enough for outcomes to occur
- Adequacy of follow-up cohorts

**NOS for case-control studies**

Selection

- Is the case definition adequate?
- Representativeness of the cases
- Selection of controls
- Definition of controls

Comparability of cases and controls on the basis of the design or analysis (worth 1 or 2 stars)

Exposure

- Ascertainment of exposure
- Same method of ascertainment for cases and controls
- Non-response rate

**NOS for cross-sectional studies**

Selection

- Representativeness of the cases
- Non-respondents
- Ascertainment of the exposure (risk factor)

Comparability of the participants based on the design or analysis (worth 1 or 2 stars)

Outcome

- Assessment of the outcome
- Statistical tests

**Thresholds for overall risk of bias judgments in RoB-2**

- Low risk of bias: The study is judged to be at low risk of bias for all domains for this result
- Some concerns: The study is judged to raise some concerns in at least one domain for this result, but not to be at high risk of bias for any domain
- High risk of bias: The study is judged to be at high risk of bias in at least one domain for this result, or the study is judged to have some concerns for multiple domains in a way that substantially lowers confidence in the result.

**Thresholds for overall risk of bias judgments in ROBINS-I**

- Low risk of bias: The study is judged to be at low risk of bias for all domains
- Moderate risk of bias: The study is judged to be at low or moderate risk of bias for all domains
- Serious risk of bias: The study is judged to be at serious risk of bias in at least one domain, but not at critical risk of bias in any domain
- Critical risk of bias: The study is judged to be at critical risk of bias in at least one domain
- No information: There is no clear indication that the study is at serious or critical risk of bias and there is a lack of information in one or more key domains of bias (a judgment is required for this)
