# Supplementary material for: It’s all in the music: A systematic review on the effects of musical characteristics on participants’ experience and behavior during leisure activities
Source: PLoS One. 2025 Jul 22;20(7):e0315986. doi: 10.1371/journal.pone.0315986 (PMC12282921; doi:10.1371/journal.pone.0315986)
Supplement: S3 File — (DOCX) [file pone.0315986.s003.docx]

## Original YANS questionnaire

1. I think that the sound level at discos, dances, rock concerts and sporting events, in general, is too loud. (F1)
2. Listening to music while doing homework helps me concentrate. (F2)
3. I am prepared to do something to make the school environment quieter. (F4)
4. I consider leaving a disco, rock concert, dance or sporting event if the sound level is too loud. (F1)
5. I can concentrate even if there are many different sounds around me. (F2)
6. I think it is unnecessary to use earplugs when I am at a disco, rock concert, dance or sporting event. (F1)
7. It is important for me to make my sound environment more comfortable. (F4)
8. I don’t like when it is quiet around me. (F2)
9. The sound level at discos, dances, rock concerts or sporting events is not a problem. (F1)
10. Noise and loud sounds are natural parts of our society. (F1)
11. Traffic noise is not disturbing. (F3)
12. The sound level should be lowered at discos, rock concerts, dances or sporting events. (F1)
13. I think it should be quiet and calm in the classroom. (F4)
14. Sounds from fans, refrigerators, computers, etc., do not disturb me. (F3)
15. I am prepared to give up activities where the sound level is too loud. (F1)
16. The sound level at my school is comfortable. (F3)
17. It is easy for me to ignore traffic noise. (F3)
18. There should be more rules or regulations for the sound levels in society. (F1)
19. When I cannot get rid of sounds that bother me, I feel helpless. (F4)
