# Supplementary material for: It’s all in the music: A systematic review on the effects of musical characteristics on participants’ experience and behavior during leisure activities
Source: PLoS One. 2025 Jul 22;20(7):e0315986. doi: 10.1371/journal.pone.0315986 (PMC12282921; doi:10.1371/journal.pone.0315986)
Supplement: Attachment — Submitted filename: Response to Reviewers.docx [file pone.0315986.s005.docx]

Dear editor,

We would like to thank the reviewers for their comments on our manuscript entitled “It’s all in the music: a systematic review on the effects of musical characteristics on participants’ experience and behavior during leisure activities”.

Our detailed responses to the reviewers’ comments can be found below.

Thank you for considering our manuscript for publication.

Sincerely,

On behalf of the author-team,

Céline Daelemans

| **Academic editor:** | **Response** |
| --- | --- |
| When submitting your revision, we need you to address these additional requirements.  Please ensure that your manuscript meets PLOS ONE's style requirements, including those for file naming. The PLOS ONE style templates can be found at  https://journals.plos.org/plosone/s/file?id=wjVg/PLOSOne_formatting_sample_main_body.pdf and  https://journals.plos.org/plosone/s/file?id=ba62/PLOSOne_formatting_sample_title_authors_affiliations.pdf | The following items have now been adapted to the PLOS ONE style requirements: headings, in-text citations of figures, supporting information citation style, captions, and file naming. |
| As required by our policy on Data Availability, please ensure your manuscript or supplementary information includes the following:  A numbered table of all studies identified in the literature search, including those that were excluded from the analyses.  For every excluded study, the table should list the reason(s) for exclusion.  If any of the included studies are unpublished, include a link (URL) to the primary source or detailed information about how the content can be accessed. | We have now uploaded to the Zenodo data repository a new table with all articles extracted from our search. The exclusion reasons for each excluded article can also be found on that table.  Name of the file on Zenodo: extracted_ articles_table_Oct 2024 |
| As required by our policy on Data Availability, please ensure your manuscript or supplementary information includes the following:  A table of all data extracted from the primary research sources for the systematic review and/or meta-analysis. The table must include the following information for each study:  Name of data extractors and date of data extraction  Confirmation that the study was eligible to be included in the review.  All data extracted from each study for the reported systematic review and/or meta-analysis that would be needed to replicate your analyses.  If data or supporting information were obtained from another source (e.g. correspondence with the author of the original research article), please provide the source of data and dates on which the data/information were obtained by your research group. | A new version of our excel sheet containing our extracted data was uploaded on Zenodo. The “data extraction information tab” contains: the name of each data extractors, the date of data extraction, confirmation that the study was eligible to be included in the review, and the source (and date) of any data that was obtained from a source other than the published article. All data extracted from each study can be found in the other tabs.  Name of the file on Zenodo: Data extraction_tabular data_November 2024_2.0 |
| As required by our policy on Data Availability, please ensure your manuscript or supplementary information includes the following:  If applicable for your analysis, a table showing the completed risk of bias and quality/certainty assessments for each study or outcome. Please ensure this is provided for each domain or parameter assessed. For example, if you used the Cochrane risk-of-bias tool for randomized trials, provide answers to each of the signalling questions for each study. If you used GRADE to assess certainty of evidence, provide judgements about each of the quality of evidence factor. This should be provided for each outcome. | All the risks of bias analysis are presented in the tab “risk of bias assessment” in our data extraction table. All of the domains assessed for the Newcastle-Ottawa Quality Assessment scale (NOS) can be found directly in the data extraction table. Regarding the RoB 2/RoB 2 for crossover trials and the ROBINS-I, their overall results can be found in the data extraction table, but the forms with all the domains completed can be found on Zenodo.  Name of the files on Zenodo:  Data extraction_tabular data_November 2024_2.0  Risk_of_bias_RoB 2_crossover_randomized_trials.docx  Risk_of_bias_ROBINS-I_non-randomized_studies.docx |
| As required by our policy on Data Availability, please ensure your manuscript or supplementary information includes the following:  An explanation of how missing data were handled. | We have clarified our approach for handling missing data in lines 177-181. The quoted texts used to replace missing data can be found in the table with all extracted data, which is published in the Zenodo data repository. |
| **Reviewer 1:** | **Response** |
| Line 126: does the inclusion of lab-based studies clash with your introduction, results and discussion? | We understand the concern that the inclusion of lab-based studies might appear to conflict with the stated aim of our review, which is to explore the behaviour and experiences of individuals attending leisure activities. However, as now clarified in both the methods (lines 147–152) and results sections (lines 280–284), we only included lab-based studies that demonstrated a high degree of ecological validity—specifically those conducted in real or purpose-built environments such as research performance halls or bars designed to closely resemble actual leisure settings.  Studies involving virtual simulations or restrictive environments, where participants could not behave as they normally would in a real-world setting, were excluded on the basis of insufficient ecological validity. In contrast, the included lab-based studies provided participants with the opportunity to engage freely and authentically in the activity being replicated. Therefore, we consider their findings to be representative of typical leisure activity experiences, despite the research context. |
| Line 137: did you conduct the search only on that day? Was it repeated at other times? Who performed the search? | Thank you for your question. Our initial search, performed on 19 October 2023, had to be repeated due to time constraints within our research group, which extended the writing process and delayed our first submission to the journal. As a result, the PLOS ONE editorial office informed us that an updated literature search was required to meet their publication criteria. Accordingly, we repeated the search on 24 October 2024. The data in this review are based on that last search.  In response to your remarks, we have now provided additional details about the literature search in lines 162–164 of the revised manuscript. |
| Pag 20 and 36 are difficult to read as tables overlap the text. Please fix it. Please place the tables in a separate document. | We apologize for the inconvenience. However, according to PLOS ONE submission guidelines, tables must be included in the main text immediately following the paragraph in which they are first cited, and cannot be submitted as a separate file.  We have positioned the tables to new pages at the end of the corresponding sections to minimize overlap with the text. We anticipate that the final published version, formatted in the PLOS ONE online layout, will resolve any remaining readability concerns. |
| Line 588-561: I appreciate your protocol but these factors should be discussed. | We appreciate the reviewer’s comment and have added clarification to the manuscript. The rationale for selecting age, hearing health history, and frequency of attendance as comparability factors is now explained in the methods (lines 207-209), and their impact on the interpretation of findings is addressed in the discussion (lines 643-650). While these factors are important potential confounders, they were inconsistently reported across studies and varied widely, contributing to substantial heterogeneity. As a result, it was not scientifically appropriate to pool outcomes or directly compare studies, in line with the exploratory nature of our review. |
| Line 54: has been gaining in popularity -> has gained popularity. | Thank you for your correction, we have now changed it to “has gained popularity”. |
| Line 58: please add a reference for identified as a growing threat. | Thank you for your remark, we have added a reference for the WHO as they are our source for the identified growing threat. |
| Line 64: “permitted” and “up to” are a bit odd. Maybe: enabled these audio devices to reach maximum volumes of 78 to 136 dB. | We have removed this sentence in accordance with the reviewers' other suggestions regarding the introduction. |
| Line 72: please add also notch HL. | We have added notch hearing loss in the sentence. |
| Line 81: the sentence “some European country … ones” is too vague. | Thank you for your remark, we have corrected this by specifying the number of European countries which were mentioned in the referenced article as having a well-developed jurisdiction on noise levels, and have cited 3 of those countries as an example. |
| Line 84: the campaigns should be in quotes. | We have added quotation marks for each of the three campaigns. |
| Line 88: maybe add Dolan - Preferred music-listening level in musicians and non-musicians. | Thank you for the recommendation. We fully agree that the article by Dolan & Kluk is relevant in this context and have now cited it in lines 96-98. |
| Line 112: thank you for the checklist. | We have referred to the checklist that is in our supporting information. |
| Line 186: did you encounter any discrepancies in assessing the risk of bias? If so, how were they resolved? | There were only a few minor discrepancies between the reviewers during the risk of bias assessment. These were promptly discussed and resolved between the two reviewers.  We have now clarified this in the manuscript. |
| Line 224: remove [21], no need of it. | We have removed the reference for this sentence. |
| Line 225: please mention the papers with 16 and 3256 participants. | We have now added the article reference after each of the two cited sample sizes. |
| Line 228: “The most common” should be in a new line. | We have now placed “The most common” on a new line as requested. |
| Line 228-229: remove bracket. | The brackets were removed and the sentence was adjusted accordingly |
| Line 231: to improve readability, please list the 9 categories as a bulleted or numbered list. | Thank you for the suggestion. We agree that using bullet points will enhance the readability, particularly since it is important for the reader to clearly understand which outcomes were extracted from the included articles. This formatting will also help the reader to more easily follow our results section. |
| Line 347: clarify the YANS and YANS-R abbreviations and add references. | We have clarified the abbreviations for YANS (Youth Attitude to Noise Scale) and YANS-R (Revised Youth Attitude to Noise Scale) in lines 391-397, added the corresponding references [60, 56], and provided further explanation on how both questionnaires work. Additionally, the full original YANS questionnaire has been included in the supplementary material (S3 File) for reference. |
| Line 349: since it’s the only significant value, please add which test and its results. | Thank you for the comment. This sentence refers to the preceding one and aims to convey that seven out of the eight studies using the YANS or YANS-R questionnaires reported statistically significant results. We chose to report a range of values rather than listing each study’s result individually, as including all details in the text would be too lengthy. Instead, we direct the reader to Table 7 for the exact values.  However, we acknowledge that this was not clearly communicated in the original manuscript and have rephrased the sentence to enhance clarity. |
| Line 351-355: if the sentences are citations, they should be in quotes. | The results by Carter & Black and Welch & Fremaux were not previously extracted as direct citations; however, this has been revised, and we have therefore added quotation marks. |
| Line 377-379: please rephrase | We have reorganised the sentence so that each numerical value is now clearly positioned in front of the corresponding music condition, ensuring clarity. |
| Line 385: “Due to the extensive data, the detailed results were not included in Table 8.” should be a footnote. | Footnotes are not permitted in the PLOS ONE submission guidelines, which is why we have added it to the main text. |
| Pag 36: it’s not clear what the numeric adverbs refer to. | Thank you for your helpful suggestion. We have revised the paragraph to remove the numerical adverbs (e.g., firstly, secondly, etc.) in order to improve readability and flow. Additionally, we have restructured the text so that each sub-outcome now appears at the beginning of its respective sentence or paragraph, making the organization and content clearer for the reader. |
| Line 418: here and elsewhere p<0.01, being constant how do you report results please. | We agree with your comment and have now followed these guidelines when reporting p-values: exact values were provided in all cases, except for very small p-values, where we used the threshold of *p* < 0.001. |
| Line 429-430: please change how you report CI as double minus is confusing. | We agree that the use of double minus signs can be confusing. Therefore, we have replaced the dashes (previously used to present the confidence intervals) with the word 'to' for greater clarity. |
| Line 451: either comma or period. | We apologize for the typo; the comma was not intended and has therefore been removed. |
| Line 454: 9.5 out of? | The data has been corrected, as the number of aggression incidents was mistakenly extracted as the mean across both venues instead of the total. Following this correction, we revised the text as suggested and now indicate that 19 of the 487 clients observed at the hardcore venues were involved in aggression incidents. |
| Line 463: p<0.03 is odd | This p-value was taken directly from the article of interest. We did not perform the t-test ourselves and therefore cannot provide an alternative p-value. Since it is not <0.001, we have reported it in its exact form in accordance with the guidelines we have chosen to follow. |
| Line 465: euro? | Yes, the authors of this article used euros as the currency to measure the bars’ mean turnover. To improve clarity, we rephrased the sentence and replaced the word 'euros' with the symbol '€'. |
| Line 469: maybe a better title would be along the line of hearing and HPD outcomes | Thank you for the suggestion. We fully agree with the proposed title and have updated it accordingly. |
| Line 472-475: please rephrase as it’s not clear. | We have revised this section to improve clarity by rephrasing and restructuring the sentences. The updated version now presents the findings more clearly and concisely. |
| Line 478: please elaborate “numerous hearing health issues” | We have expanded this section to include more detailed information on the outcomes and results reported by Zocoli et al. |
| Line 483: first time BAHPHL used in the text please clarify the abbreviation and add references. | We have now written out the full name of the BAHPHL questionnaire at its first mention in the text, and included the corresponding reference. |
| Line 560: “The poor” should be in a new line. | Thank you for the suggestion, we have introduced it as a new line as recommended. |
| **Reviewer 2 :** | **Response** |
| The manuscript offers an extensive review of the literature regarding the factors contributing to hearing damage caused by exposure to elevated sound levels. However, the primary aim of the study is to analyze how different musical characteristics—such as tempo, tonality, rhythm, and sound level—affect individuals' behavior and experience in recreational settings.  My main concern is that the thematic transition between hearing damage and the analysis of musical characteristics is not well articulated, resulting in a lack of cohesion in the structure of the introduction. Although a strong context is provided on the negative effects of high noise levels, the manuscript seems to focus on the impact of musical characteristics on listeners, yet this topic is not adequately developed in this section.  The excessive emphasis on hearing damage may divert the reader’s attention from the true purpose of the study. Furthermore, the introduction lacks a precise explanation of which musical characteristics will be analyzed in detail and a clear justification for their selection.  I recommend that the authors expand the introduction, offering a more detailed argument about the musical characteristics to be discussed, their interaction with the sound environment, and their impact on the recreational experience. This would provide a stronger conceptual framework aligned with the study's objectives, aiding readers’ understanding from the outset. | Thank you for your helpful comments and suggestions. We agree that the introduction lacked coherence with our research question, and we have made the following adjustments accordingly:   - The sections discussing current sound levels, the negative effects of high noise exposure, and prevention methods have been shortened. However, we believe it is important for readers to understand these hearing health consequences, as they are part of our study outcomes. We have also retained sufficient detail on existing prevention strategies, as they provide relevant context for the potential real-life applications of our review. - We have improved the transition from the discussion of high sound levels to other musical characteristics by expanding on the various factors that influence musical satisfaction from the participants’ perspective. - We have introduced several musical characteristics that will be assessed, along with a brief overview of their potential impact on the audience’s experience. As our inclusion criteria are intentionally broad, we are unable to elaborate further on the rationale behind the selection of each characteristic. However, we have clarified the inclusion and exclusion criteria and provided additional explanation regarding the categorization of these characteristics. |
| In the data collection section (lines 150-151), the authors state that "Only interventions and outcomes which were relevant to the research question were extracted." However, the exact research question is unclear, making it difficult to understand the strategy used to select the articles. It is crucial that the research question(s) be explicitly stated at the end of the introduction, along with the study's hypotheses, if applicable. This would provide greater clarity regarding the study’s direction and would more precisely justify the selection of the articles included in the review. | We have now clearly stated our research question at the end of the introduction. However, we do not believe that including specific hypotheses is necessary or appropriate for this review. Given the broad and exploratory nature of our research question, formulating precise hypotheses would be limiting and could misrepresent the scope and intent of our work. |
| In line 551, the authors mention, "The broad research questions of this review allowed for several relationships between musical characteristics and participants’ experiences and behavior to be drawn. However, it also allowed for studies with very heterogeneous data to be included, making it difficult to attain clear conclusions" While it is understandable that the diversity of studies reviewed may complicate deriving solid conclusions, it is essential for the authors to further elaborate on how this heterogeneity might have impacted the interpretation of the findings.  In particular, the lack of validity in many of the reviewed studies (e.g., design issues, biases, and the absence of comparable data) raises serious doubts about the reliability of the conclusions. If the foundation on which this review is built is methodologically flawed, it becomes challenging to assess the robustness of the claims presented. | Thank you for your insightful comment. We agree that the difficulty to attain a clear conclusion is limited by the heterogeneous nature of the included studies and data. To address your concern, we now explained more clearly in lines 613-624 that while this heterogeneity limited the ability to draw overarching conclusions or pool data from studies, it enabled us to formulate recommendations for future studies on this topic. These specific findings, when considered together, contribute to a more nuanced and comprehensive understanding of our overall research question.  We also appreciate the reviewer’s concern regarding the validity and reliability of conclusions in light of the high risk of bias present in several included studies. We have now addressed this more explicitly in the manuscript (lines 625-629) by acknowledging that although the risk of bias limited some aspects of interpretation, our domain-based approach allowed us to mitigate this limitation by considering findings within their specific context. |
| Additionally, in line 560, the authors acknowledge that "The poor study design of some studies further limited the conclusions", but they do not sufficiently develop how this fact compromises the credibility of the results obtained. It is imperative to discuss in greater depth the relationship between these methodological limitations and the extracted results, as these issues could significantly impact the review's ability to generate reliable and useful conclusions. The reader should be aware of how these shortcomings condition the interpretation of the findings. | Thank you for your valuable remark. In a revised paragraph lines 629-641, we have now thoroughly expanded on the four articles identified as having the highest risk of bias and discussed in detail how these methodological limitations may affect the reliability and interpretation of our conclusions. We would also like to refer you to our earlier response, where we explain that while some specific conclusions may be limited by these issues, the overall objective and scope of our review remain unaffected. |
| Furthermore, in line 545, the authors suggest that "According to our results, specific music genres were associated with an increase in aggression, sexual assault incidence, and substance consumption". While this is an intriguing point, there is a lack of clarity regarding the stance venue owners should take in response to these findings. Although the authors acknowledge that musical characteristics, besides volume, influence attendees' experience and behavior, they do not offer clear recommendations for venue owners. It would be highly beneficial if the authors included more specific suggestions, such as which musical genres could minimize aggressive behavior or substance abuse without negatively impacting the musical experience for clients. These recommendations would add practical and tangible value to the study's findings. | We agree that it would be interesting to provide specific recommendations to venue owners. However, we believe that such recommendations would be too speculative based on the current review, as we did not examine the effects of direct interventions. What our review does highlight is the potential for future research into how other musical characteristics could be leveraged for behavioral interventions. We have clarified this point in lines 608-612 and indicated which music genres were associated with negative impacts on customer behavior in line 604 . |
| The current title, "Temporary fun, permanent damage," suggests a direct relationship between fun and permanent damage, which is neither demonstrated nor thoroughly discussed in the review. This statement could lead to misinterpretations of the study’s results and conclusions. A more appropriate title would focus on the topics that are actually addressed, namely, the effects of musical characteristics on participants' experience and behavior during recreational activities. I recommend adjusting the title to more accurately reflect the study's focus and scope, avoiding claims not supported by the evidence presented. | We appreciate your suggestion and have now changed the title to “ It’s all in the music: a systematic review on the effects of musical characteristics on participants’ experience and behavior during leisure activities”. |
| Page 5, last paragraph:  The study’s objective is mentioned twice in the last paragraph of the introduction. | This has been removed and replaced by the research question, which is now clearly stated in the final paragraph of the introduction. |
| Page 6, line 105:  The authors state, "Understanding the influence that musical characteristics can have on one’s experience at events can aid policymakers in navigating around the public’s opinion to create guidelines for a safer yet equally entertaining experience". In this case, I believe the phrase requires more clarity regarding how exactly this balance between auditory safety and pleasurable experience for attendees could be achieved. The concept of "balancing public opinion" is vague, and it is unclear how musical characteristics relate to the formation of public policy in this context. It would be useful to specify how data on musical characteristics could be concretely used to influence policymakers' decisions, especially regarding setting volume limits or regulations on the type of music and its impact on auditory health. This would help connect the research more directly to practical and policy applications. | Thank you for your insightful comment. We agree that the original statement lacked clarity. In response, we have revised the paragraph to better specify how the data presented in this review could inform the use of musical characteristics—beyond just sound levels—as a means to lower preferred listening levels at leisure events. This approach could provide policymakers with alternative strategies for reducing auditory health risks without compromising the quality of the musical experience. To illustrate this, we have added an example regarding low frequencies, which have been shown in prior research to reduce listeners' preferred sound pressure levels. This concrete example helps demonstrate how musical characteristics could be leveraged in future policy or regulatory frameworks aimed at safer sound environments. |
| Page 7, line 150:  The authors mention: "If these effect measures were not reported in the article according to the interventions of interest, the corresponding authors were contacted to obtain the raw data. " It would be advisable to specify how many cases involved contacting authors and how many of these resulted in effective responses. | Thank you for your suggestion, we have added the recommended information in lines 182-183. |
| Page 9, line 190:  In the section on effect measures, the authors state: "If these effect measures were not reported in the article according to the interventions of interest, corresponding authors were contacted for raw data." It would be helpful to specify how many cases involved contacting authors for raw data and how many attempts resulted in obtaining the required information. | Thank you for your suggestion, we have added the recommended information in lines 225-226.. |
| Page 10, line 210:  The authors state: "After the full texts of the articles were retrieved and assessed for eligibility, 44 were excluded mainly due to wrong outcomes or designs, and others for alternative reasons (see Figure 1)" It would be helpful for the authors to specify the criteria used to determine that these articles had "incorrect designs." | We have now clarified what we mean by "wrong study design" in the Methods section (lines 142–148). However, it is important to note that "wrong study design" was used as a broad exclusion category due to the wide variety of articles retrieved during our literature search. We determined that creating highly specific exclusion labels for each study would result in an overly complex and fragmented set of categories. Therefore, this label was applied to studies that did not align with our methodological framework—for instance, those that reversed our intended independent variables and outcomes, or lacked appropriate controls for musical characteristics. We hope this explanation provides greater transparency regarding our exclusion criteria. |
| It would be advisable for the authors to consider condensing some of the results presented in tables into figures, as this would facilitate a quicker and clearer understanding of the findings for readers. Figures, such as graphs or diagrams, can highlight patterns, trends, and comparisons more visually and accessibly than tables. Additionally, figures allow relationships between different variables to be captured at a glance, expediting the interpretation process without sacrificing data precision. | We have discussed this matter within our research group and decided not to present our findings in figures. We believe that using figures may oversimplify the results and increase the risk of drawing incorrect conclusions about trends and interpretations. Furthermore, since our study includes a large amount of data across multiple datasets that do not share the same control variable, we believe that summary tables are more appropriate for presenting this type of data, as outlined in the article by Franzblau and Chung.  Source: Franzblau, L.E. and Chung, K.C. (2012) ‘Graphs, tables, and figures in scientific publications: The good, the bad, and how not to be the latter’, *The Journal of Hand Surgery*, 37(3), pp. 591–596. doi:10.1016/j.jhsa.2011.12.041. |
