# Supplementary material for: It’s all in the music: A systematic review on the effects of musical characteristics on participants’ experience and behavior during leisure activities
Source: PLoS One. 2025 Jul 22;20(7):e0315986. doi: 10.1371/journal.pone.0315986 (PMC12282921; doi:10.1371/journal.pone.0315986)
Supplement: Attachment — Submitted filename: Response_to_Reviewers_auresp_2.docx [file pone.0315986.s006.docx]

Dear editor,

We would like to thank the reviewers for their comments on our manuscript entitled “It’s all in the music: a systematic review on the effects of musical characteristics on participants’ experience and behavior during leisure activities”.

Our detailed responses to the editor and reviewers comments can be found below.

Thank you for considering our manuscript for publication.

Sincerely,

On behalf of the author-team,

Céline Daelemans

| **Academic editor** | **Response** |
| --- | --- |
| Please review your reference list to ensure that it is complete and correct. If you have cited papers that have been retracted, please include the rationale for doing so in the manuscript text, or remove these references and replace them with relevant current references. Any changes to the reference list should be mentioned in the rebuttal letter that accompanies your revised manuscript. If you need to cite a retracted article, indicate the article’s retracted status in the References list and also include a citation and full reference for the retraction notice. | We have reviewed our reference list and removed an unpublished article to comply with PLOS ONE’s guidelines. Because the removed article related to the YANS questionnaire, we replaced it with the published source from which the complete questionnaire included in our supplementary files (S3) was extracted.  Additionally, we have verified using Zotero reference manager software that none of our currently cited papers have been retracted.  Summary of changes to the reference list:   - Removed unpublished article : Olsen SE, Erlandsson S. I. Youth Attitude to Noise Scale (YANS). Licentiate dissertation (unpublished). University of Goteborg, 2004) - Added published article as a replacement for the YANS questionnaire: Zocoli AMF, Morata TC, Marques JM. Youth Attitude to Noise Scale (YANS) questionnaire adaptation into Brazilian Portuguese. Braz J Otorhinolaryngol 2009;75:485–92. https://doi.org/10.1016/S1808-8694(15)30484-5 - Removed Reference 26 due to the removal of the quality column in table 2 and 3 according to the reviewer’s comment below: Penson DF, Krishnaswami S, Jules A, Seroogy JC, McPheeters ML. Evaluation and Treatment of Cryptorchidism. Comparative Effectiveness Reviews,. 2012. |
| **Reviewer 1** | **Response** |
| Lines 92-93: “several studies” but only one was mentioned. Furthermore, several of the statements of the introduction are based on [2] Beach and Gilliver “Time to listen”. Please find other sources to corroborate some of them. | We have added two additional references to corroborate the statement in lines 92–93. Furthermore, we have included supplementary sources for most of the statements previously citing only Beach and Gilliver's “Time to Listen. |
| Line 115: remove “low” | Has now been removed |
| Line 119: in participants of leisure → among participants; affect → influence | Thank you for your suggestion, we have now adapted our research question according to your comment. |
| Lines 186-199: “The extraction form was composed of eight sections.” but only four sections are clearly marked. | We have removed the numeric adverbs from this paragraph, as they previously caused confusion by implying there were only four sections. All eight sections are now clearly cited: design details, arms, arm details, sample characteristics, outcomes, outcome details, results, and risk of bias assessment. |
| Line 244: “wrong outcomes”. It’s wrong for your aim, not for their studies. Please change the adjective. | We have revised our sentence to clarify that the outcomes and study designs were excluded because they did not meet our inclusion criteria. The adjective "wrong" was replaced with "unrelated." |
| Fig 1: as above, change “wrong”. | We will upload a new Fig 1 with the adjectives for outcome and design updated to "unrelated" and "non-conforming," respectively. |
| Line 283 and Table 1: “We did not specifically note in Table 1 that these studies were conducted in lab settings”. I disagree. I think this information is needed for comprehensiveness. | We have now added a note “(simulated in a lab)” in the leisure activity column of Table 1 for each article that used an ecologically valid laboratory setting. We would like to clarify that most of these “laboratories” are frequently used as concert venues with no research conducted during these events. |
| Lines 288-289: please add reasoning why you only included low groove/low tempo. In the results (line 429) and Table 8 you mentioned also high groove and tempo. | We apologize for the misunderstanding. Lines 288–289 actually do not state that only the low groove/low tempo group was studied. Instead, they explain that this condition served as the control group from which we derived our calculations presented in the results section. Since this appeared to cause confusion, we have clarified the sentence and moved it to lines 374-375 |
| Table 1: several studies did not report age. How confident are you that the study did not include participants <15 years old? | We understand your concern. To address your question clearly, we have added explanations for each study in Table 1 that does not explicitly state a mean age:   - Dolan et al. 2018: The paper specifies 22 adults. - Eichwald et al. 2023: The authors state their population consisted exclusively of adults. - Engels et al. 2011: The study was conducted in a bar (alcohol cannot legally be served to individuals under 18 in the Netherlands), and the authors indicate their participants were mostly university students. - Forsyth, 2009: The study was conducted in nightclubs in Glasgow city center, where minors under 18 are prohibited. Thus, it is extremely unlikely more than 50% of participants were under 15 years old. - Gueguen et al. 2008: Included 40 males aged 18 to 25. Exact ages were not provided, so a mean could not be calculated. - Gueguen et al. 2004: Participants were bar patrons, making it improbable that more than 50% of participants were under 15 years old. - Kayser et al. 2021: Participants were recruited via word-of-mouth, mailing lists, and leaflets at the University of York, making it unlikely for individuals younger than 15 years old to participate. Additionally, minors under 15 would have required parental consent, which the authors would likely have mentioned. - Mahomed et al. 2024: Participants were explicitly described as being between 18 and 26 years old. Exact individual ages were unavailable for mean calculation. - Merrill et al. 2023: While the exact ages were not stated, participant educational levels strongly suggest that none were below 15 years old. Most held university degrees or equivalent qualifications. - Welch & Fremaux, 2017: The authors explicitly state participants were between 18 and 26 years old. Exact individual ages were unavailable for mean calculation. |
| Lines 300-301, 303-304, 311-312, and 316-317: remove new lines. | Thank you for pointing this out. The issue occurred during the conversion of our manuscript from DOCX to PDF. Specifically, extra lines above each table caused unwanted paragraph breaks when the tables were cited in the Risk of Bias section. We have fixed this by removing those lines. |
| Tables 2 and 3: Remove the column “quality” as it was not in the NOS. The list as table note isn’t easy to digest. Similar for the other tables. | We have removed the “quality” column in table 2 and 3 and any text describing the thresholds for converting the NOS into the AHRQ standards of quality. To make the footnotes clearer we have moved all the risk of assessment criteria to a supplementary file so that less text is present in footnotes. Furthermore we have also tried to shorten the footnotes by making running sentences when possible instead of new paragraphs everytime. |
| Line 393-395 : no need of F1, F2 etc. later factor 1 → first factor. | The text has been adapted according to your suggestion. |
| Line 403: “Black”. Remove full stop. | Done |
| Table 7 and others: For the notes, please use the conventional symbol order so readers don’t need to count the number of asterisks. See <https://en.wikipedia.org/wiki/Note_(typography)#Numbering_and_symbols> | In response to your comment, we have revised the footnote symbols to follow the conventional order, as outlined in the referenced source. |
| Table 8: what VLF, PC1, and PC2 stand for? | We have added the full forms of the abbreviations below Table 8. |
| Lines 453 and 455: I appreciate the precision in reporting the p-values but using “p>0.05” would improve the flow. | We have revised the in-text reporting of p-values in accordance with your comment. |
| Line 484: which other two articles? | The other two articles have been cited for clarification. |
